# A case of infantile Barth syndrome with severe heart failure: Importance of splicing variants in the *TAZ* gene

**DOI:** 10.1002/mgg3.2190

**Published:** 2023-04-25

**Authors:** Atsuhito Takeda, Masahiro Ueki, Jiro Abe, Kazuhiro Maeta, Tomoko Horiguchi, Hirokuni Yamazawa, Gaku Izumi, Ayako Chida‐Nagai, Daisuke Sasaki, Takao Tsujioka, Itsumi Sato, Masahiro Shiraishi, Masafumi Matsuo

**Affiliations:** ^1^ Department of Pediatrics, Faculty of Medicine Hokkaido University Sapporo Japan; ^2^ MRC Mitochondrial Biology Unit University of Cambridge Cambridge UK; ^3^ KNC Department of Nucleic Acid Drug Discovery, Faculty of Rehabilitation Kobe Gakuin University Kobe Japan; ^4^ Research Center for Locomotion Biology Kobe Gakuin University Kobe Japan; ^5^ Faculty of Health Sciences Kobe Tokiwa University Kobe Japan

**Keywords:** Barth syndrome, cardiomyopathy, left ventricular noncompaction, minigene, splicing variants

## Abstract

Barth syndrome (BTHS) is an X‐linked disorder characterized by cardiomyopathy, skeletal myopathy, and 3‐methylglutaconic aciduria. The causative pathogenic variants for BTHS are in *TAZ*, which encodes a putative acyltransferase named tafazzin and is involved in the remodeling of cardiolipin in the inner mitochondrial membranes. Pathogenic variants in *TAZ* result in mitochondrial structural and functional abnormalities. We report a case of infantile BTHS with severe heart failure, left ventricular noncompaction, and lactic acidosis, having a missense c.640C>T (p.His214Tyr) variant in *TAZ*, which is considered a pathogenic variant based on the previously reported amino acid substitution at the same site (c.641A>G, p.His214Arg). However, in this previously reported case, heart function was compensated and not entirely similar to the present case. Silico prediction analysis suggested that c.640C>T could alter the *TAZ* messenger RNA (mRNA) splicing process. *TAZ* mRNAs in isolated peripheral mononuclear cells from the patient and in vitro splicing analysis using minigenes of *TAZ* found an 8 bp deletion at the 3′ end of exon 8, which resulted in the formation of a termination codon in the coding region of exon 9 (H214Nfs*3). These findings suggest that splicing abnormalities should always be considered in BTHS.

## Graphical abstract

We report a case of infantile Barth syndrome with severe heart failure and lactic acidosis, having a missense c.640C>T (p.His214Tyr) variant in TAZ gene. Silico prediction analysis suggested that c.640C>T could alter the TAZ mRNA splicing process. TAZ mRNAs and in vitro splicing analysis using minigenes of TAZ found an 8 bp deletion at the 3′ end of exon 8, which resulted in the formation of a termination codon in the coding region of exon 9 (H214Nfs*3). These findings suggest that splicing abnormalities should always be considered in Barth syndrome.

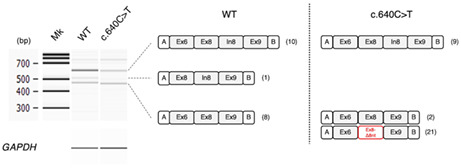

## 1 INTRODUCTION

Barth syndrome (OMIM #302060) (BTHS) is an X‐linked lipid metabolism disorder characterized by cardiomyopathy, skeletal myopathy, neutropenia, growth delays, and 3‐methylglutaconic aciduria. Among cardiomyopathies, left ventricular noncompaction (LVNC), characterized by prominent left ventricular trabeculae and deep intertrabecular recesses, is particularly characteristic of BTHS. The causative pathogenic variants for BTHS are in *TAZ*, which encodes a putative acyltransferase named tafazzin and is involved in the remodeling of cardiolipin (CL) in the inner mitochondrial membranes (Takeda et al., 2011). Pathogenic variants in *TAZ* cause an accumulation of monolysocardiolipin and reduced levels of tetralinoeoyl CL, which result in mitochondrial structural and functional abnormalities. To date, more than 200 pathogenic variants have been reported. Although a genotype–phenotype correlation has not yet been established, pathogenic truncating variants always result in a serious clinical course and are often fatal. Therefore, it is important to determine whether the missense variant is related to splicing abnormalities. Here, we report a severe infantile BTHS case in which a missense variant in *TAZ* shows various splicing variants, including pathogenic truncated variants, which are considered to have contributed to the worsening of the clinical course.

## 2 CASE REPORT

**FIGURE 1:**
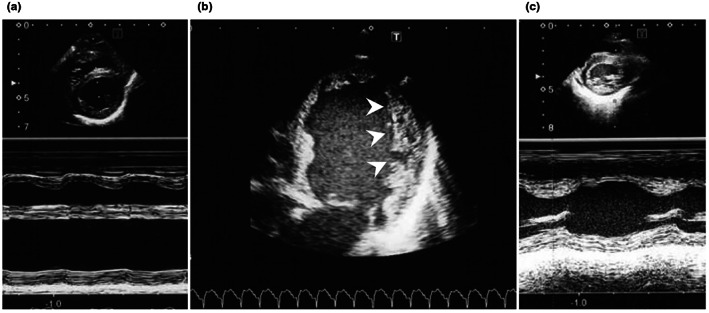
Echocardiographic findings. (a) M‐mode echocardiography from a parasternal left ventricular short‐axis view on admission. The left ventricular diameter was dilated (left ventricular end‐diastolic diameter was 150% of normal) and cardiac pump function was markedly reduced (20% of left ventricular ejection fraction). (b) B‐mode echocardiography from an apical four chamber view. Prominent left ventricular trabeculae and deep intertrabecular recesses (arrowheads) were found. The ratio between the noncompacted and compacted layers was calculated to be 2.0 at the lateral side of the left ventricular wall at the end‐diastolic phase, which meets the criteria for left ventricular noncompaction. (c) M‐mode echocardiography from a parasternal left ventricular short‐axis view 2 months after admission. The left ventricular diameter was reduced (left ventricular end‐diastolic diameter was 116% of normal) and cardiac pump function was improved (left ventricular ejection fraction 55%).

The patient was born via spontaneous delivery to nonconsanguineous parents at 39 weeks of gestation. The APGAR score was 9 at both 1 min and 5 min. After birth, the patient had no symptoms of heart failure. Two weeks after birth, the patient was transferred to our hospital because of severe heart failure with metabolic crisis. No other symptoms, including muscle weakness or hematological abnormalities such as neutropenia, were observed. Echocardiography showed a dilated left ventricular size (150% of normal for left ventricular end‐diastolic diameter) with severely decreased cardiac pump function, where the left ventricular ejection fraction was 20% (Figure 1a). Furthermore, prominent left ventricular trabeculae and deep intertrabecular recesses met the criteria for LVNC (Figure 1b). Blood gas analysis showed marked metabolic acidosis (pH 6.89, base excess −23.4 mmol/L, and lactate 126 mg/dL), suggesting an inborn error of metabolism including mitochondrial disorders. Together with LVNC, BTHS was strongly suspected and genetic testing for *TAZ* was performed. According to the local ethics committee recommendations of Hokkaido University Hospital, isolation of genomic DNA and molecular analysis of *TAZ* were performed for the patient and the patient's mother (to identify an X‐linked disorder) after informed consent had been obtained for both the patient and his mother. Hemizygous C to T substitution (c.640C>T, p.His214Tyr) was found within exon 8 of *TAZ* in the patient. The same mutant signal was detected in the mother in a heterozygous form, indicating that she is the carrier. Ferri et al. reported a BTHS case having a pathogenic missense variant in *TAZ* with an amino acid substitution at the same site (c.641A>G, p.His214Arg) (Ferri et al., 2013). In this patient, LVNC and metabolic acidosis were both present at birth; however, cardiac function was compensated for, and there were no heart failure symptoms throughout the neonatal and infantile period. Conversely, in our case, heart function was decompensated, leading to severe heart failure, which was apparently different in terms of cardiac function severity.

**FIGURE 2:**
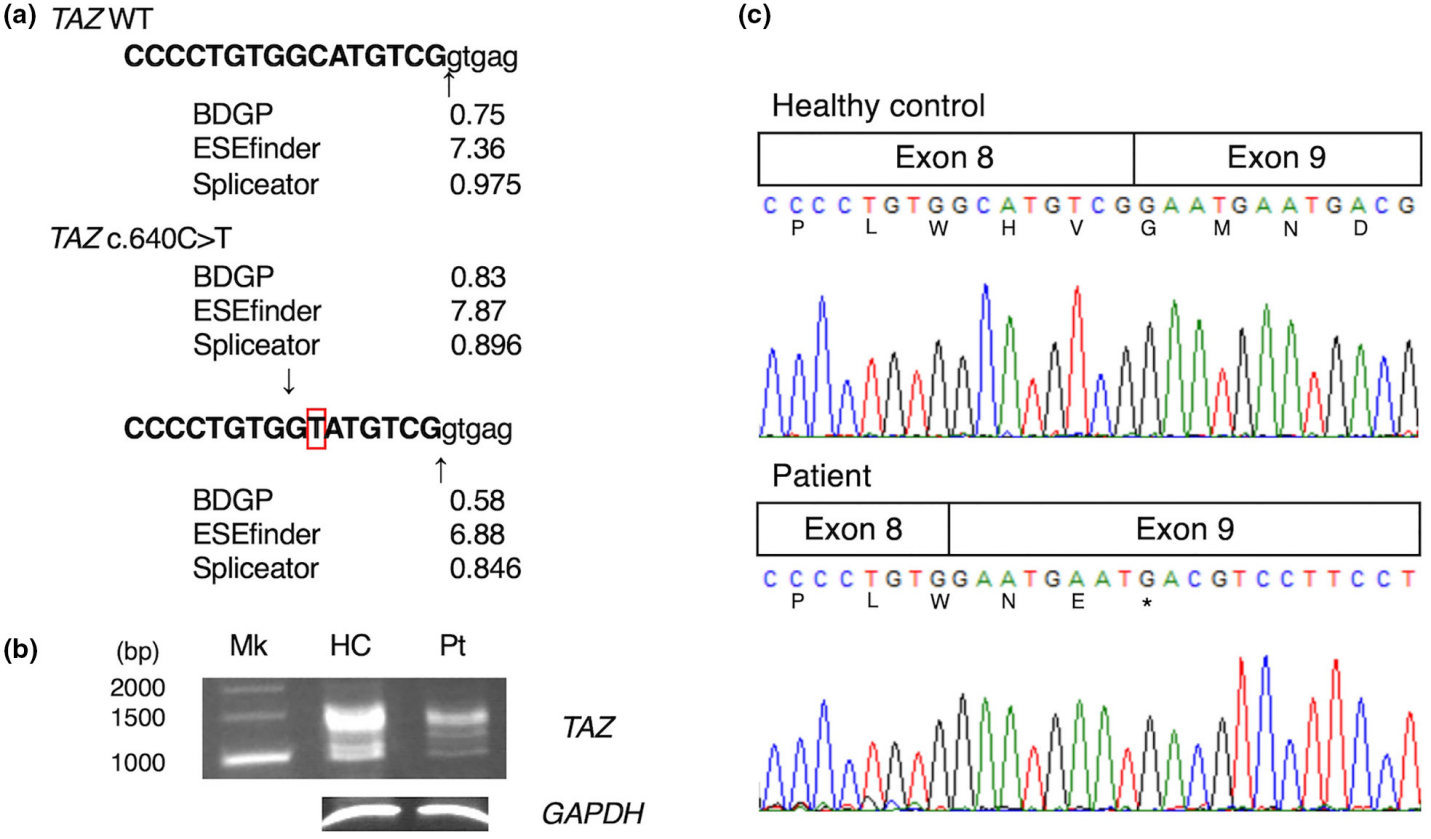
Altered splice site by c.640C>T substitution in the patient. (a) Scores calculated by the splice site prediction software (Berkeley Drosophila Genome Project (NNSPLICE 0.9 version), ESEfinder 3.0, or Spliceator 2.1) in *TAZ* wild‐type (WT) and c.640C>T substitution. (b) RT‐PCR amplification of full‐length *TAZ* cDNA from a healthy control (HC) and the patient (Pt). Electropherograms of the amplified products are shown with size markers on the left (Mk). *GAPDH* cDNA was amplified as a control. (c) Sequence analysis of *TAZ* cDNA around c.640C>T substitution in healthy control and the patient. Nucleotide sequences and their translated amino acid sequences are shown. The 8‐nucleotide deletion in exon 8 causes a frameshift variant, which alters the amino acids and derives a termination codon (*) in the coding region of exon 9.

In our patient, a vitamin cocktail therapy for metabolic crisis had a significant effect on acute heart failure, and cardiac function gradually improved after the introduction of cardioprotective therapy including carvedilol and enalapril (Abe et al., 2020) (Figure 1c). Our patient is now 2 years old, not strong enough to walk alone, but doing well without symptoms of heart failure. Thus far, there has been no evidence of intermittent neutropenia or 3‐methylglutaconic aciduria. Based on our case, we considered the possibility of a splicing abnormality rather than a simple amino acid substitution at p.His214 because the heart failure was more severe than those of a previously reported case with a substitution at p.His214Arg. The c.640C>T gene variant in exon 8 of *TAZ* was not reported in gnomAD nor Togo VAR and resulted in the newly formed GT dinucleotide that could be a donor site for splicing. The predicted score of splicing using ESEfinder 3.0 (Scalzitti et al., 2021) (ESE), Berkeley Drosophila Genome Project (BDGP; NNSPLICE 0.9 version) (Wang et al., 2014), and Spliceator 2.1 (Reese et al., 1997) suggested that the GT dinucleotide generated by the c.640C>T could be a new splice donor site, and all the values of these scores for the new splicing site (BDGP 0.83, ESE 7.87, and Spliceator 0.896) were higher than normal (BDGP 0.58, ESE 6.88, and Spliceator 0.846) (Figure 2a). To test this hypothesis, we analyzed the messenger RNA (mRNA) of isolated peripheral mononuclear cells (PBMCs) from whole blood of the patient using reverse transcription‐polymerase chain reaction (RT‐PCR) and performed in vitro splicing analysis using minigenes of *TAZ*.

## 3 MATERIALS AND METHODS

### 3.1 Isolation of PBMCs, extraction of RNA, and generation of complementary DNA

Procedures were performed as previously described (Yamada et al., 2012). Briefly, PBMCs were separated from the heparinized blood from the patient or healthy controls by density gradient using Histopaque (Sigma‐Aldrich, St. Louis, MO, USA). RNA was extracted from PBMCs using Trizol Reagent (Thermo Fisher Scientific, Waltham, MA, USA) following the manufacturer's instructions. Complementary DNA (cDNA) was generated from mRNA using random primers by PrimeScript RT Reagent kit (Takara Bio Inc., Shiga, Japan).

The primers amplifying the *TAZ* cDNA (NM_000116.5) and sequencing *TAZ* exon 7–10 were designed using Primer BLAST (Ye et al., 2012). The primers used were TAZcDNAF: 5′‐gaggtcgcagacctagaggc‐3′ and TAZcDNAR: 5′‐ ggaggagctggaatgcctac‐3′ (SeqF: 5′‐aagctcaaccatggggactg‐3′ SeqR: 5′‐ccgacttgttctccgccc‐3′). Sequence analysis was performed using Applied Biosystems 3730xl DNA Analyzer at FASMAC Co. Ltd. (Atsugi, Japan).

### 3.2 Minigene construction

**FIGURE 3:**
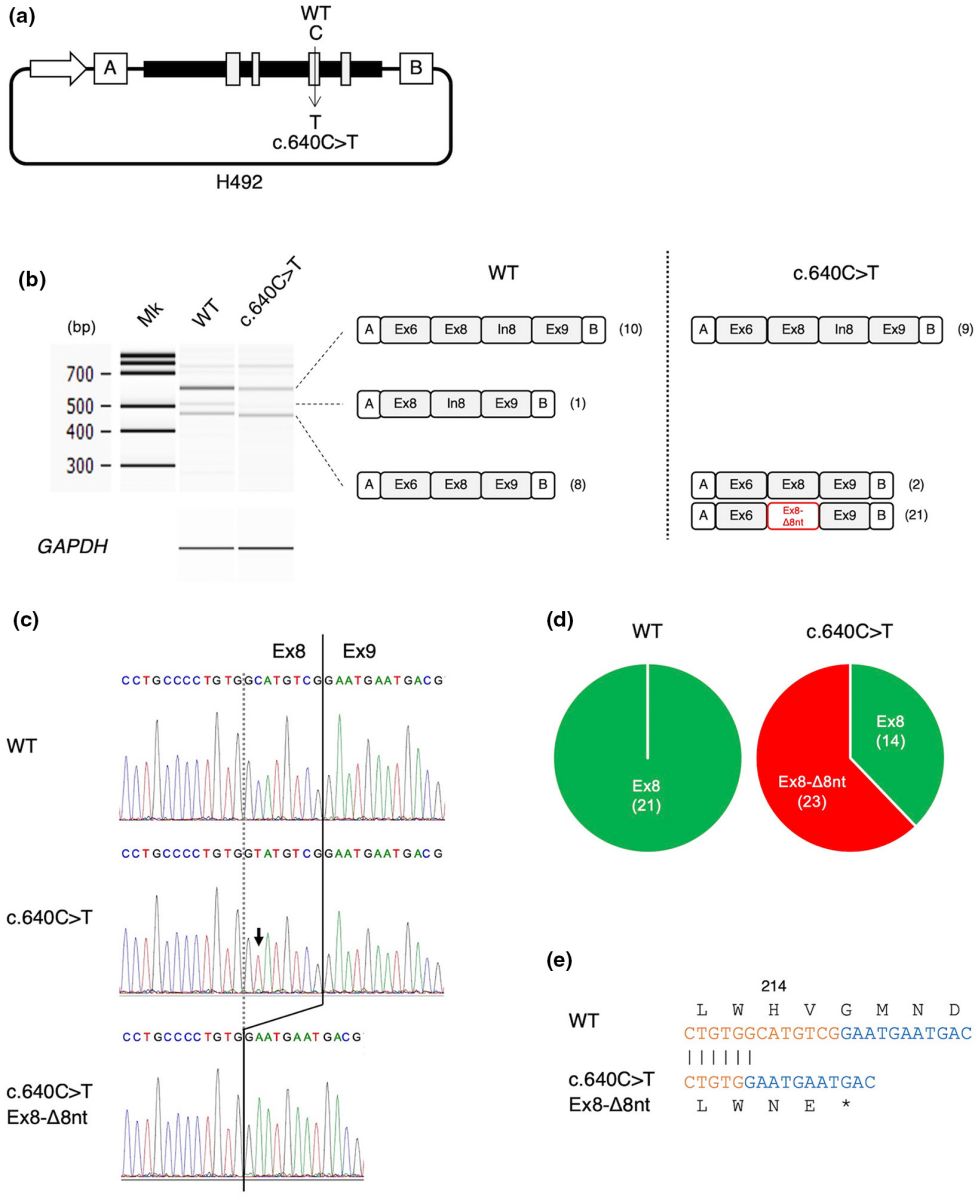
Splicing analysis of 640C>T *TAZ* minigene. (a) Schematic of a minigene plasmid containing exons 6, 7, 8, and 9 (gray boxes) and introns 5, 6, 7, 8, and 9 (black boxes) in either H492TAZEx6‐9 (wild‐type [WT]) or H492TAZEx6‐9 m (c.640C>T). The c.640C>T is in exon 8. The minigene vector H492 contains two cassette exons (A: DMD exon 18 and B: DMD exon 20, white boxes) and an intron sequence containing a multi‐cloning site. These plasmids were introduced into human umbilical vein endothelial cells (HUVEC) cells and the pre‐mRNA was transcribed from the cytomegalovirus (CMV) promoter (white arrow). (b) RT‐PCR amplification of minigene in HUVEC cells. Electropherograms of the amplified products are shown with size markers on the left (Mk). *GAPDH* mRNA was also amplified as a control. The structure of *TAZ* variants is shown schematically. Boxes indicate exons and introns. The Ex8‐Δ8nt (red) indicates the 8‐nucleotide deletion of exon 8. A total of 21 and 37 splicing products were subcloned from H492TAZEx6‐9 (WT) and H492TAZEx6‐9 m (c.640C>T), respectively. The number in parentheses indicates the number of clones obtained by TA cloning. (c) Sequence at the junction of exons 8 and 9. The arrow indicates the c.640C>T (middle panel). Eight nucleotides (GTATGTCG) at the 3′ end of exon 8 were missing in the splicing product of the c.640C>T (lower panel). (d) Ratio of 8‐nucleotide deletion in all clones of TA cloning. The number of each clone is shown in parentheses. (e) Amino acid sequences were translated from the novel transcripts. Nucleotide sequences (exon 8: orange, exon 9: blue) and their translated amino acid sequences are shown. The 8‐nucleotide deletion in exon 8 causes a frameshift variant, which alters the amino acids and derives a termination codon (*) in the coding region of exon 9. Numbers indicate amino acid residues in tafazzin (NP_000107.1).

To construct H492TAZEx6‐9, a fragment containing exons 6, 7, 8, and 9 and the adjacent intronic regions were amplified from genomic DNA (BioChain Institute, Newark, CA, USA) by PCR. The primers used were TAZIn5NheIF: 5′‐gcggctagcgaggaatggtggaggctgag‐3′ and TAZIn9BamHIR: 5′‐gcgggatccgatgaggagcagtaggagtgcc‐3′. The amplified product was digested with NheI and BamHI (New England Biolabs, JP) and inserted into a ready‐made splicing assay minigene H492 (Habara et al., 2009), which was digested with the same restriction enzymes. To construct H492TAZEx6‐9 m, which contains the *TAZ* c.640C>T in exon 8, site‐directed mutagenesis was introduced into H492TAZEx6‐9 using the PrimeSTAR Mutagenesis Basal Kit (Takara Bio Inc.) (Figure 3a). The primers used in this study were TAZc640CTF: 5′‐atcatcctgcccctgtggtatgtcgg‐3′ and TAZc640CTR2: 5′‐ccccaggctcaccgacataccacagg‐3′. All the plasmids stated above were confirmed by sequence analysis.

### 3.3 Cell culture and transfection

Human umbilical vein endothelial cells were purchased from Takara Bio Inc. (C‐12206). Cells were cultured in Endothelial Cell Growth Medium 2 Kit (Takara Bio Inc.) at 37°C in a 5% CO_2_ humidified incubator. For transfection, 1.5 × 10^5^ cells grown in 12‐well culture plates were incubated with 3 μL of Lipofectamine LTX Reagent (Thermo Fisher Scientific), 3 μL of PLUS Reagent, and 1 μg of plasmid. RNA was extracted from the cells 24 h after plasmid transfection.

### 3.4 In vitro splicing analysis

In vitro splicing analysis was performed using H492TAZEx6‐9 and H492TAZEx6‐9 m. RNA was extracted using the High Pure RNA isolation kit (Roche Diagnostics, Basel, Switzerland). cDNA was synthesized from 0.5 μg of each RNA using random primers (Thermo Fisher Scientific) and M‐MLV Reverse Transcriptase (Thermo Fisher Scientific). The transcripts were PCR amplified using primers (T7‐F: 5′‐taatacgactcactataggg‐3′ and BGH‐R: 5′‐tagaaggcacagtcgagg‐3′). The integrity of the cDNA was examined by amplifying the mRNA of the glyceraldehyde 3‐phosphate dehydrogenase (*GAPDH*) gene using a set of primers on exons 3 and 6 (GAPDH H_F: 5′‐cccttcattgacctcaac‐3′ and GAPDH H_R: 5′‐ttcacacccatgacgaac‐3′). PCR was performed with 2 μL of cDNA, 2 μL of 10× ExTaq buffer, 0.25 U of ExTaq polymerase (Takara Bio Inc.), 500 nM of each primer, and 200 μM of dNTPs at a total volume of 20 μL. For the amplification of transcripts, 30 cycles of amplification were performed in a Mastercycler Gradient PCR (Eppendorf, Hamburg, Germany) under the following conditions: initial denaturation at 94°C for 3 min, denaturation at 94°C for 0.5 min, annealing at 60°C for 0.5 min, and extension at 72°C for 1.5 min. For the amplification of *GAPDH*, 18 cycles of amplification were performed. The amplified PCR products were electrophoresed and semi‐quantified on an Agilent 2100 Bioanalyzer (Agilent Technologies, Santa Clara, CA, USA) using the DNA 1000LabChip kit.

### 3.5 DNA sequencing

For the analysis of minigene splicing products, PCR products were gel extracted with QIAquick Gel Extraction Kit (Qiagen, Hilden, Germany) and subcloned in pT7Blue T‐vector (Novagen, Madison, WI, USA) using DNA Ligation kit Ver. 2.1 (Takara Bio Inc.). The subcloned insert was sequenced by Applied Biosystems 3730xl DNA Analyzer at FASMAC Co. Ltd. (Atsugi) using two primers (YH307: 5′‐attactcgctcagaagctgtgttgc‐3′ and YH308: 5′‐aagtctctcacttagcaactggcag‐3′) for exons A and B.

## 4 RESULTS

The sequence of full‐length *TAZ* cDNA from the patient demonstrated that bands were slightly weak compared with the healthy control (Figure 2b), and an 8 bp deletion at the 3′ end of exon 8 was found in the patient (Figure 2c). In vitro splicing analysis using minigenes of *TAZ* showed several splicing variants (Figure 3a,b). A total of 21 and 37 clones were obtained from the splicing products of H492TAZEx6‐9 and H492TAZEx6‐9 m, respectively, as a result of TA cloning (Figure 3b). All clones yielded amplified products containing exons A and B. The wild‐type (WT) minigene splicing product showed a normal splicing pattern between exons 8 and 9 (Figure 3c, upper panel). However, the splicing product of the c.640C>T mutant minigene revealed an 8 bp deletion at the 3′ end of exon 8 (Figure 3c, lower panel). The percentage of clones with the 8 bp deletion in exon 8 was 0% for the WT minigene splicing product and 62% for the c.640C>T mutant minigene splicing product (Figure 3d).

## 5 DISCUSSION

LVNC is an important causative disease of heart failure and has been associated with pathological variants in almost 20 different genes. Defects in sarcomere genes, such as *MYH7*, *MYBPC3*, *TNNT2*, *TPM1*, *ACTC1*, and *TTN* have been reported as causative for LVNC and LVNC is also commonly associated with mitochondrial diseases (Gerull et al., 2019). When LVNC is associated with lactic acidosis, as in the present case, mitochondrial diseases are highly likely, and *TAZ* should be considered a causative gene, particularly in males. Although there have been many reports on pathological variants in *TAZ*, the relationship between genotype and phenotype, especially the severe form, is not well understood. Thus, we focused our study on the splicing variants. Using mRNA analysis from patient PBMCs and minigene analysis, we confirmed that the c.640C>T causes an 8 bp deletion at the 3′ end of exon 8 in *TAZ*, which is consistent with the results predicted by the splicing scores. This may cause a frameshift in the amino acid reading frame, resulting in the formation of a termination codon in the coding region of exon 9 (H214Nfs*3, Figure 3e). Contrarily, if the splicing site is the same as normal, no frameshift would occur and a missense variant in which histidine 214 mutates to tyrosine would be expected (Figure 3c, middle panel). A patient carrying a c.641A>G in *TAZ*, LVNC with compensated cardiac function, has a missense variant in which histidine 214 is replaced by arginine. This suggests that the missense variant in histidine 214 is less symptomatic than the 8 bp deletion at the 3′ end of exon 8 found in our patient.

Kirwin et al. reported the presence of multiple alternative splice variants in *TAZ* mRNA even in controls and reported that pathogenic variants may result in more complex alternative splicing patterns (Kirwin et al., 2014). Ferri et al. reported a case of BTHS, a severe dilated cardiomyopathy in infancy with a synonymous variant (C.348C>T p.Gly116Gly), and found by mRNA analysis that the amino acid deletions were caused by splicing abnormalities (Ferri et al., 2016).

These findings suggest that amino acid substitutions associated with missense variants in *TAZ* alone are not sufficient to explain the disease severity, and that splicing abnormalities should be predicted and mRNA and minigene analyses be performed if needed.

Furthermore, in gene therapy, splicing can be artificially controlled to return the splicing site to the normal donor site by the promotion of tafazzin expression, which has a single amino acid substitution but is close to normal (Matsuo, 2021).

In summary, we report a case of severe infantile BTHS in which a missense variant in *TAZ* exhibited various splicing variants and contributed to the deterioration of the clinical course. We concluded that it is important to determine whether missense variants are related to splicing abnormalities whenever possible.

## AUTHOR CONTRIBUTIONS

Atsuhito Takeda, Masahiro Ueki, and Masafumi Matsuo conceived and planned the experiments. Atsuhito Takeda, Masahiro Ueki, Kazuhiro Maeta, Tomoko Horiguchi, and Masafumi Matsuo carried out the experiments. Atsuhito Takeda, Jiro Abe, Hirokuni Yamazawa, Gaku Izumi, Ayako Chida‐Nagai, Daisuke Sasaki, Takao Tsujioka, Itsumi Sato, and Masahiro Shiraishi contributed to clinical data preparation. Atsuhito Takeda, Masahiro Ueki, Kazuhiro Maeta, Tomoko Horiguchi, and Masafumi Matsuo wrote the manuscript. All authors discussed the results and commented on the manuscript.

## ETHICS STATEMENT

None.

## FUNDING INFORMATION

None.

## CONFLICT OF INTEREST STATEMENT

KM is employed by KNC Laboratories Co., Ltd., Kobe, Japan. MM discloses being employed by Kobe Gakuin University, which received funding from KNC Laboratories Co., Ltd., Kobe, Japan. MM further discloses being a scientific adviser for Daiichi‐Sankyo Co., Tokyo, Japan, and JCR Pharma Co., Ashiya, Japan. The other authors declare that they have no competing interests.

## Data Availability

The data that support the findings of this study are available from the corresponding author upon reasonable request.
